# Emerging Issues on Antibiotic-Resistant Bacteria Colonizing Plastic Waste in Aquatic Ecosystems

**DOI:** 10.3390/antibiotics13040339

**Published:** 2024-04-08

**Authors:** Ifra Ferheen, Roberto Spurio, Stefania Marcheggiani

**Affiliations:** 1 School of Biosciences and Veterinary Medicine, University of Camerino, 62032 Camerino, Italy; ifra.ferheen@unicam.it (I.F.); roberto.spurio@unicam.it (R.S.); 2 Department of Environment and Primary Prevention, National Institute of Health, 00161 Rome, Italy

**Keywords:** multidrug-resistant bacteria, *Staphylococcus* spp., *Klebsiella* spp., antibiotic resistance genes, aquatic ecosystem, One-Health

## Abstract

Antibiotic-resistant bacteria (ARB) adhesion onto plastic substrates is a potential threat to environmental and human health. This current research investigates the prevalence of two relevant human pathogens, *Staphylococcus* spp. and *Klebsiella* spp., and their sophisticated equipment of antibiotic-resistant genes (ARGs), retrieved from plastic substrates submerged into an inland water body. The results of microbiological analysis on selective and chromogenic media revealed the presence of colonies with distinctive phenotypes, which were identified using biochemical and molecular methods. 16S rDNA sequencing and BLAST analysis confirmed the presence of *Klebsiella* spp., while in the case of *Staphylococcus* spp., 63.6% of strains were found to be members of *Lysinibacillus* spp., and the remaining 36.3% were identified as *Exiguobacterium acetylicum*. The Kirby–Bauer disc diffusion assay was performed to test the susceptibility of the isolates to nine commercially available antibiotics, while the genotypic resistant profile was determined for two genes of class 1 integrons and eighteen ARGs belonging to different classes of antibiotics. All isolated bacteria displayed a high prevalence of resistance against all tested antibiotics. These findings provide insights into the emerging risks linked to colonization by potential human opportunistic pathogens on plastic waste commonly found in aquatic ecosystems.

## 1. Introduction

Antibiotic resistance is an inevitable consequence of the selection pressure exerted on bacteria, and it may swiftly develop and spread in bacteria that adhere to surfaces or form biofilms [1]. Bacterial biofilms consist of a population of sessile microorganisms, which exhibit a substantial increase in resistance to antibiotics, reaching levels up to 1000 times higher than their individual planktonic state, a phenomenon that poses a major barrier in treating and eradicating surface-attached bacteria [2]. Biofilm formation is a crucial virulence factor, involving cell attachment, proliferation, matrix production, and cell separation. Bacterial attachment can occur on biotic and abiotic environmental surfaces and on medical devices such as intravascular catheters, endotracheal tubes, and prosthetic implants. Biofilms can hinder host immune mechanisms and the activity of antimicrobials and disinfectants, leading to persistent and chronic bacterial infections. The epidemiological risks of biofilm-associated infections have been reported from all continents in recent years and the health issues associated with pathogenic bacteria such as *Staphylococcus aureus* and *Klebsiella pneumoniae* are becoming the subject of public awareness campaigns and debate [3,4,5].

One rarely explored feature of plastic debris in aquatic ecosystems is their potential to act as a resilient substrate for biofilm formation and a long-lasting surface for exchanging genetic information [6,7]. Plastic pollution in aquatic ecosystems poses a severe threat to planetary health as commercial plastic production has increased drastically over the last 60 years, reaching levels of up to 300 million tons per year, and it may soon surpass the total fish biomass in aquatic ecosystems [8]. The transmission of plastic-associated antibiotic-resistant bacteria (ARB) in aquatic ecosystems is an emerging issue, and the link between plastic pollution, the spread of pathogenic bacteria, and human health problems is widely reported in the literature [9,10].

The role played by plastics in marine ecosystems as vehicles of pathogenic bacteria has been deeply examined, but only a few studies have investigated their presence in inland waters and the possible consequences for animal and human health [11,12]. Furthermore, since different plastic polymers find their way into the aquatic ecosystem, their inherent physical and chemical properties—such as hydrophobicity, charge, and surface roughness—may have an influence on the adherence and proliferation of bacteria. There is a lack of research studies on the interactions between certain harmful bacteria and different plastic polymers. Therefore, this work aimed to examine the ability of two potentially pathogenic bacteria, *Klebsiella* spp. and *Staphylococcus* spp., to adhere to four plastic polymers commonly present as plastic waste in aquatic ecosystems and to investigate their contribution to the transmission of antibiotic resistance genes.

## 2. Results

### 2.1. Isolation of Targeted Bacteria

This study was conducted across seven geographical locations around a Caldera-type Lake in the Latium Region of Italy, in November 2021. Four plastic polymers were submerged in water for 30 days to foster the proliferation of *Staphylococcus* spp. and *Klebsiella* spp. Thirty-one bacterial strains from cultivation media displayed the characteristic phenotypes along the *Staphylococcus* and *Klebsiella* genera.

**Figure 1:**
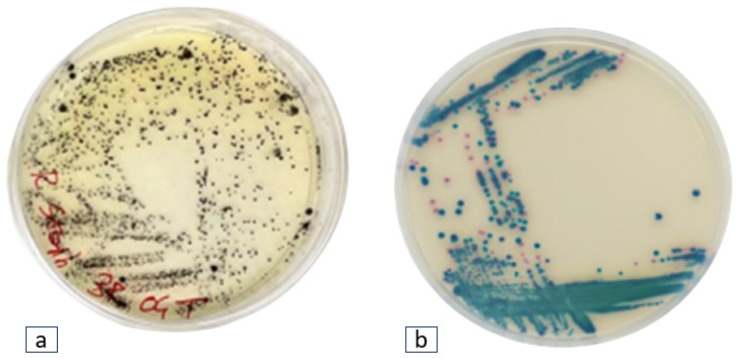
Isolation of targeted bacteria from plastic polymers: (**a**) Black colonies depicting typical phenotype of *Staphylococcus* spp. on RAPID-Staph Agar. (**b**) Green-blue colonies of *Klebsiella* spp. on ESBL agar medium.

Twenty-two black colonies with halo zones exhibiting phenotypic traits attributed to *Staphylococcus* spp., and nine blue-green colonies indicative of *Klebsiella* spp. were found (Figure 1).

### 2.2. Identification of Targeted Bacteria

Following the microbiological screening, the thirty-one bacterial strains preliminary identified as *Staphylococcus* spp. and *Klebsiella* spp. underwent biochemical identification by using API-Staph and API-20E kits, respectively. The biochemical test results indicate the 22 potential *Staphylococcus* isolates corresponded to the following species: *Staphylococcus intermedius* (*n* = 4), *Staphylococcus captions* (*n* = 2), *Staphylococcus haemolyticus* (*n* = 3), *Staphylococcus xylosus* (*n* = 2), *Staphylococcus lentus* (*n* = 2), *Staphylococcus sciuri* (*n* = 4), and *Staphylococcus aureus* (*n* = 5). Meanwhile, the nine isolates obtained from the ESBL medium were identified as *Klebsiella pneumoniae* (*n* = 5) and *Klebsiella oxytoca* (*n* = 4).

To validate the results derived from biochemical identification, strains underwent further classification based on 16S rDNA amplification and sequencing. BLAST analysis revealed that among the *Staphylococcus* spp. strains (*n* = 22), 36.3% were identified as *Exiguobacterium acetylicum*, while 63.6% were classified as various species of *Lysinibacillus*, notably *Lysinibacillus fusiformis* (*n* = 5), *Lysinibacillus boronitolerans* (*n* = 3), *Lysinibacillus sphaericus* (*n* = 4), and *Lysinibacillus macrolides* (*n* = 3). Regarding *Klebsiella* spp., 16S rDNA sequencing confirmed the identification of *Klebsiella pneumoniae* (*n* = 4) and *Klebsiella oxytoca* (*n* = 2). Additionally, three isolates were identified as *Klebsiella michiganensis*, which were not detected via biochemical identification. A summary of the twenty isolates identified by biochemical and molecular identification is provided in Table 1, while some of the isolates have already been described [13].

Some molecular identification results diverged from those obtained through standard biochemical methods. It is well known that biochemical kits were designed for the rapid identification of clinical strains to provide a prompt therapeutic response. However, they may exhibit a high probability of yielding false-negative or false-positive results when used against environmental bacterial strains [14]. Thus, while preliminary screening and identification can be conducted via biochemical methods, the validation of results necessitates molecular-based techniques [15,16]. Consequently, strains biochemically identified as various species of *Staphylococci* were reclassified as *Exiguobacterium* and *Lysinibacillus* species, underscoring the importance of 16S rDNA sequencing in mitigating any potential false-positive outcome.

### 2.3. Colonization of Bacteria on Different Plastic Polymers

**Figure 2:**
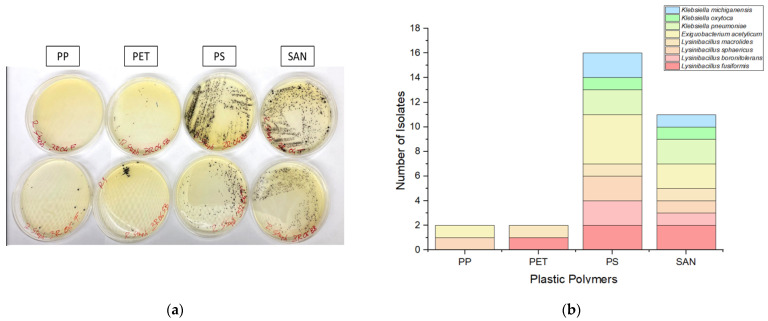
(**a**) The isolation of *Staphylococcus* spp. from four distinct plastic polymers on Rapid-Staph agar (duplicate samples) revealed a higher prevalence of black colonies on polystyrene (PS) and styrene acrylonitrile resin (SAN) compared to polypropylene (PP) and polyethylene terephthalate (PET), indicating differential adherence patterns among the polymers. (**b**) The number of targeted bacteria isolated from each plastic polymer illustrates the bacterial adhesion trend as being PS > SAN > PET ≈ PP.

In this study, we investigated the adherence of targeted bacteria to four prevalent plastic polymers frequently encountered in aquatic ecosystems, namely polyethylene (PE), polypropylene (PP), polyethylene terephthalate (PET), and styrene acrylonitrile resin (SAN). A notable imbalance in bacterial adhesion among these polymers was observed, with a higher number of bacteria attached to polystyrene (PS) and styrene acrylonitrile resin (SAN) surfaces compared to polypropylene (PP) and polyethylene terephthalate (PET). Specifically, the adherence trend observed was PS > SAN > PP ≈ PET, indicating that the intrinsic properties of different plastic polymers significantly influence bacterial colonization (Figure 2).

Interestingly, PP and PET were found to harbor only a limited number of bacterial species, comprising *Lysinibacillus* spp. and *Exiguobacterium acetylicum*, with no isolates of *Klebsiella* identified. In contrast, PS and SAN exhibited greater effectiveness as substrates, fostering a broader spectrum of bacterial colonization. These substrates supported the growth of diverse bacteria, including *Exiguobacterium acetylicum*, *Lysinibacillus fusiformis*, *Lysinibacillus boronitolerans*, *Lysinibacillus sphaericus*, *Lysinibacillus macrolides*, *Klebsiella oxytoca*, *Klebsiella pneumoniae*, and *Klebsiella michiganensis*.

These results, which outline the differential ability of bacteria found in aquatic ecosystems to colonize plastic polymers with varying chemical properties, were aligned with the findings of Alshehrei et al., who similarly reported that various plastic polymers offer diverse colonizing sites due to differences in their chemical composition, zeta potential, surface morphology, surface charge, hydrophobic properties, and morphology [17]. Hydrophobic surfaces tend to promote bacterial adhesion due to the preferential attraction of hydrophobic regions on bacterial cell surfaces [18]. A report from Cai et al. revealed that surfaces with more hydrophobic features favor bacterial colonization owing to their property of inhibiting hydrogen bridges formed by water molecules [19]. Therefore, highly hydrophobic polymers like PP, PS, and SAN may facilitate greater bacterial attachment compared to less hydrophobic polymers (PET). Additionally, the zeta potential and surface charge can influence electrostatic interactions between bacteria and polymer surfaces [20,21]. Overall, these intrinsic properties of plastic polymers play a crucial role in mediating bacterial adhesion and colonization, thereby impacting the persistence and potential spread of bacterial pathogens in aquatic ecosystems. These polymers are commonly used in making single-use plastic materials; for instance, PP is utilized in food packaging and household items, while PET is commonly found in beverage bottles and food packaging. PS is used in disposable food service items and packaging for electronics and household goods [21], while SAN, a copolymer plastic used in place of PS due to its greater thermal resistance, is ideal for use across a range of industry applications, like disposable lighters, auto gauge covers, cosmetic cases, and medical syringes. Considering their short lifespan and the potential to evade recycling processes, plastic waste materials made from low- and high-density polyethylene, PP, PET, SAN, and PS have been extensively documented in aquatic ecosystems [22,23]. This highlights the urgent need for effective mitigation strategies to curb the discharge of plastic waste in aquatic ecosystems, thereby safeguarding both environmental and public health.

### 2.4. Phenotypic Antibiotic Susceptibility Profiling

**Figure 3:**
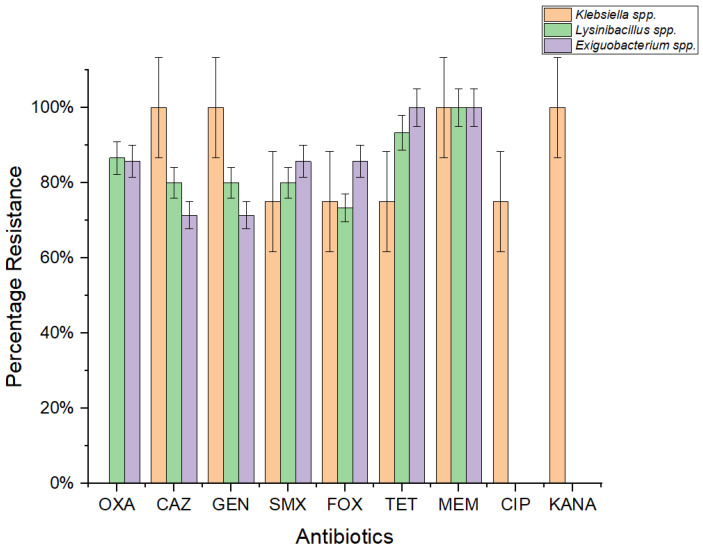
Phenotypic Antibiotics Resistance profile of *Lysinibacillus* spp., (*n* = 15) and *Exiguobacterium acetylicum* (*n* = 7) toward a panel of antibiotics active against Gram-positive bacteria, and *Klebsiella* spp. (*n* = 9) toward a panel of antibiotics effective against Gram-negative bacteria.

A diverse panel of antibiotics was employed for Gram-negative and Gram-positive bacteria. Phenotypic antibiotic susceptibility testing across a wide range of therapeutically relevant antibiotics revealed an extensive rate of resistance among *Exiguobacterium*, *Lysinibacillus*, and *Klebsiella* strains in the benthic state (Figure 3). The resistance profile of *Exiguobacterium acetylicum* indicates that all strains exhibit resistance to meropenem (MEM), while 86% of strains were resistant to oxacillin (OXA), sulfamethoxazole (SMX), and cefoxitin (FOX), and 71% demonstrated resistance to gentamicin (GEN) and ceftazidime (CAZ). In the case of *Lysinibacillus* spp., 100% of the isolates displayed resistance to MEM with decreasing values for the antibiotics TET (93%), OXA (87%), GEN (80%), CAZ (80%), and FOX (73%). Similarly, *Klebsiella oxytoca* exhibited high levels of resistance to TET, MEM, SMX, and kanamycin (KAN). Furthermore, all strains of *Klebsiella pneumoniae* demonstrated resistance to GEN, MEM, KAN, and CAZ, with 75% of the strains exhibiting resistance to CIP (ciprofloxacin), TET, SMX, and FOX. Additionally, *Klebsiella michiganensis* displayed noteworthy resistance to TET, SMX, and FOX. Overall, 67% of *Klebsiella* spp. displayed resistance against the CIP, GEN, MEM, and CAZ antibiotics, while 33% demonstrated resistance to SMX.

A study conducted at Hawassa Lake also reported high percentages of resistance of *Staphylococcus* spp. and *Klebsiella* spp. against antibiotics like norfloxacillin, ampicillin, amoxicillin, and kanamycin, highlighting the importance of aquatic ecosystems in spreading ARB [24]. Furthermore, Ochonska, Scibik, and Brzychczy-Wloch (2021) investigated *K. pneumoniae* isolated from the tracheostomy tubes of 18 patients, and reported that 44.4% of the samples had the ability to produce biofilms. Scanning electron microscopy unveiled distinct biofilms generated by strains of *K. pneumoniae* on the surfaces of polyethene (PE) and polyvinyl chloride (PVC) test tubes under laboratory conditions. Additionally, the majority of those isolates were identified as ESBL producers and harbored genes like *CTX-M-1*, *SHV*, and *TEM* [25].

### 2.5. Genotypic Antibiotic Susceptibility Profiling

Thirty-one multidrug-resistant bacteria isolated from plastic samples underwent genotypic resistance profiling for eighteen antibiotic resistance genes (ARGs) and two genes belonging to class 1 integrons (Table 2). The genotypic resistant profiling of *Lysinibacillus* spp. revealed that among 15 strains, 10 of the 18 ARGs were positively detected, including two tetracycline resistance genes (*tetA*, *tetW*), one sulphonamide resistance gene (*sul1*), four β-lactamase resistance genes (*blaCTX-M*, *blaSHV*, *blaTEM*, *mecA*), and three multidrug-resistant genes (*acrB*, *acrF*, and *adeA*). Meanwhile, strains of *Exiguobacterium acetylicum* exhibited a distinctive resistance gene pool, based on *tetA*, *blaTEM*, *cmx(A)*, *mecA*, and *adeA* genes.

On the other hand, genotypic resistance profiling of the *Klebsiella genus* revealed distinct patterns among *Klebsiella pneumoniae*, *Klebsiella oxytoca*, and *Klebsiella michiganensis*. *Klebsiella pneumoniae* demonstrated a wide spectrum of resistance genes, with notable occurrences of *blaCTX-M*, *blaSHV*, *blaTEM*, *cmx(A)*, *acrB*, *tetA*, *sul1*, and *sul2*, in contrast with *Klebsiella oxytoca*, which exhibited the significant prevalence of *blaCTX*, *blaSHV*, *acrB*, *acrR*, and *sul2* genes. Moreover, in *Klebsiella michiganensis*, a significant prevalence of *blaSHV*, *blaTEM*, *adeA*, *tetW, tetB*, *sul1*, and *acrF* was found.

In addition to ARGs, two genes encoding for class 1 integrons (*intl-1* and *intl1-V*), crucial for disseminating resistance genes among various bacterial species, were detected in all isolated strains except for *L. boronitolerans*, *L. macrolides*, and *E. acetylicum* in which *intl-1* was not detected.

## 3. Discussion

Plastic polymers are versatile and durable materials with the potential to cause diseases as several studies have reported the significant biological consequences upon the ingestion of microplastic particles [2,26]. Previous studies have suggested that these polymers, in addition to being toxic, serve as potential reservoirs for pathogenic bacteria, capable of colonizing and spreading diseases in aquatic ecosystems and beyond [13,27]. Currently, the available data reported in the literature are inconclusive regarding the extent of this phenomenon, the fluctuation metrics, and the link with anthropogenic activities [3]. The identification and characterization of antibiotic-resistant bacteria colonizing plastic waste in aquatic ecosystems represent a critical aspect for understanding the environmental dissemination of pathogens and their associated ARGs [28]. In this study, we isolated and identified potentially pathogenic bacterial strains from plastic polymers, and these findings provide empirical evidence supporting the notion that plastic waste within aquatic environments serves as a conducive environment for the attachment, proliferation, and dissemination of potential pathogenic microorganisms.

Moreover, an additional health issue associated with these bacteria is represented by their set of genes coding for antibiotic resistance determinants. The accurate detection and characterization of ARGs is a crucial step to understand and interpret the complex resistome, especially in composite environmental settings. This essential aspect of public health surveillance systems can profit from the detailed investigations carried out on specific bacteria circulating in aquatic ecosystems, combined with the know-how resulting from wide-scale metagenomic approaches, with the aim being to understand ARG distribution and transmission at the interfaces of different One-Health sectors [29].

Several studies have reported wide patterns of antibiotic resistance in the environmental isolates of potential pathogenic bacteria. According to Stankiewicz et al., *Lysinibacillus* spp. isolated from wastewater harbored β-lactamase resistance genes like *blaCTX-M*, *blaSHV*, and *blaTEM* in all samples, indicating their ability to survive in environments polluted with antibiotics [30]. The detection of *blaOXA*, *blaSHV*, *blaKPC2*, and *blaTEM* was also reported in the *Exiguobacterium* spp., *Klebsiella* spp., and *E. coli* isolated from a fresh water reservoir in Thailand [31]. The resistome of ESBL-producing *K. pneumoniae* is a collection of genes that confer antibiotic resistance to five critical classes of antibiotics commonly used in therapeutics: β-lactams, aminoglycosides, quinolones, tigecycline, and polymyxins [32]. The data from the European Antimicrobial Resistance Surveillance Network for 2005–2015 indicate the presence of non-susceptible strains of *K. pneumoniae*, *Staphylococcus aureus*, *Pseudomonas aeruginosa*, and *Escherichia coli* against significant antibiotic classes [33]. Another study, conducted on short-read sequencing of the *K. pneumoniae* KP-8b clone obtained from a PS (Styrofoam) sample found in Zanzibar coastal zones, reported the high prevalence of β-lactamases resistance genes, while another strain of *K. pneumoniae* KP-3b, obtained from a PET sample, contained sixteen distinct resistance genes making it resistant to eight different classes of antimicrobials. This highlights the severe human health consequences of infection caused by plastic-associated *K. pneumoniae* due to its ability to carry and spread multidrug resistance genes [34].

Abiotic and biotic degradation of plastics releases carbon-based substrates that are available for heterotrophic growth. A recent study reported that in surface waters, as compared to natural organic matter, the presence of plastic leachate increases biomass acquisition by 2.29 times and they support 1.72 times more efficient bacterial growth, suggesting that plastics in aquatic ecosystems foster more bacterial growth as compared to the organic matter [35]. These plastic fragments represent a new niche for microbial attachment and proliferation and provide novel hotspots for transmitting antibiotic-resistant genes via horizontal gene transfer in natural ecosystems [36]. Tons of microplastics colonized by various microorganisms including pathogenic bacteria from humans or animals in areas like wastewater treatment plants offer a significant risk of spreading antibiotic resistance. In established biofilm structures, a high bacterial density and close physical contact between cells allow bacterial transformation and conjugation, leading to the lateral transfer of mobile genetic elements (e.g., IS elements, plasmids, integrons) encoding antibiotic resistance genes (ARGs) [36,37,38]. The spread of antibiotic resistance through plastic particles could have profound consequences for the evolution of aquatic bacteria and poses a specific risk for human health.

While the present study has yielded valuable insights into the prevalence of antimicrobial-resistant (AMR) strains of potentially pathogenic bacteria on four plastic surfaces, it is essential to acknowledge limitations, to ensure a balanced interpretation of the results. The focus on selected bacterial isolates in this study may have resulted in a sample size that inadequately represents the full spectrum of antibiotic-resistant bacteria in aquatic ecosystems. The results of this work revealed the prevalence of two opportunistic human pathogens on plastic surfaces submerged in a freshwater body, but we can envisage that the whole picture is far more complex when taking into consideration the hidden world of abundant uncultivable bacteria. This scenario emphasizes the urgent need to implement effective strategies to reduce the release of pollutants into aquatic environments and to control the spread of antibiotic resistance. Hence, an interdisciplinary approach based on the collaboration of microbiologists, ecologists, and environmental engineers could provide a more comprehensive understanding of the complex network that leads to antibiotic resistance dissemination in aquatic ecosystems. The present research contributes to the implementation of the One-Health perspective regarding the role of aquatic ecosystems in transmitting pathogenic bacteria and their associated antibiotic-resistant genes.

## 4. Materials and Methods

### 4.1. Sampling and Isolation of Targeted Bacteria

**Figure 4:**
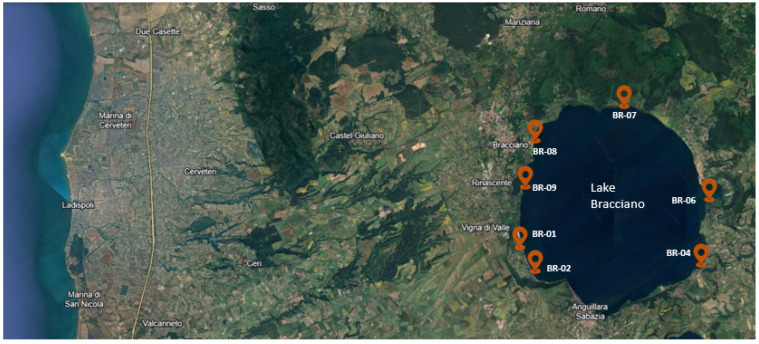
The seven geographical locations of Lake Bracciano (Italy) selected to study plastic-associated bacteria.

The current study was performed in 2021 at a lake located in central Italy (Bracciano) and involved seven geographical locations (Figure 4). The experimental setup was designed to provide a reproducible and efficient tool suitable to study the adherence and colonization of bacteria to plastic materials. Plastic pollution was simulated by using four polymers, i.e., polypropylene (PP), polystyrene (PS), styrene acrylonitrile resin (SAN), and polyethylene terephthalate (PET). The surface available for attachment was approx. 20 × 15 × 2 cm for PET and SAN (plastic box), while for PP it was 110 cm^2^ (cylindrical shape) and for PS it was 8 × 8 × 2 cm (plastic box). After one-month, plastic samples were collected from all sites, stored under dark conditions at ±4 °C, and transported to the laboratory. Further analyses were performed within 24 h of collection. The recovery from each plastic polymer of the two target bacteria (*Staphylococcus* spp. and *Klebsiella* spp.) was performed using scrapers and swabs under a laminar hood (AURA, Biosafety Cabinet Class 2).

For the microbiological analysis, the collected material was inoculated into 100 mL of pre-enrichment Mueller–Hinton broth (Oxoid, Hampshire, UK) and incubated for 24 h at 20 °C (temperature recorded at the sampling site) (Intercontinental Medical Equipment). To isolate *Staphylococcus* spp. and *Klebsiella* spp., 100 μL aliquots of pre-enriched culture were spread onto a Rapid-Staph Agar (Condalab, Madrid, Spain) and Chrome Art Extended Spectrum Beta Lactamase (ESBL) agar medium (Biomerieux, Marcy-l’Étoile, France), respectively, and incubated at 37 °C for 24 h. From each chromogenic medium, single colonies depicting the typical phenotype of targeted bacteria were isolated onto MH agar plates at the same incubation conditions mentioned above.

For further analysis, a cryobank was established for the resulting bacterial colonies following the manufacturer’s guidelines (Cryobank, Awantor, Radnor, PA, USA).

All experiments were performed in triplicate, along with positive and negative controls, and result interpretation was performed following the qualitative methodologies, i.e., the presence or absence of specific colony phenotypes.

#### 4.1.1. Identification of Targeted Bacteria

Isolates obtained from plastic samples were identified up to the genus level using the biochemical identification method. The API-Staph kit (BioMeriuex, Grassina, Italy) and API-20E kit (Biomerieux; Grassina, Italy) were used for the identification of *Staphylococcus* spp. and *Klebsiella* spp., respectively, following the manufacturer’s instructions. Each isolate was assigned a seven-digit identification code based on the positive and negative results following the API color scale. The code obtained was then entered into the APIWEB^TM^ (Biomerieux) software (https://apiweb.biomerieux.com, accessed on 1 December 2021) to identify the isolates.

Following biochemical identification, strains associated with *Staphylococcus* spp. or *Klebsiella* spp. were subjected to 16S rDNA gene amplification and sequencing, with the aim to obtain identification at the species level. Genomic DNA of targeted isolates was extracted using a GRISP DNA extraction kit (GRISP, Porto, Portugal), and the V4-V5 region of the 16S rDNA gene was amplified using primers and PCR conditions as described earlier by Ferheen et al. [7]. PCR products were purified using a PCR and Gel band Purification kit (GRISP, Porto, Portugal), and Sanger DNA sequencing was performed by Eurofins Genomics (Ebersberg, Germany).

#### 4.1.2. Antibiotic Susceptibility Test and Targeted Genotyping

The antibiotic susceptibility test (AST) of bacterial isolates was performed using the disk diffusion assay against nine commercially available antibiotics (Oxoid, Basingstoke, Hampshire, UK). Briefly, each bacterial strain was suspended in 5 mL of 0.85% saline solution to obtain 0.5 McFarland standards in turbidity, and 100 μL of this cell suspension was spread on MH agar using sterile swabs. Discs with known concentrations of antibiotics were placed on inoculated MH agar plates and incubated at 37 °C for 24 h. The concentration of antibiotic disks tested was the following: tetracycline (TET) 30 μg, gentamycin (GEN) 10 μg, chloramphenicol (CAM) 30 μg, ciprofloxacin (CIP) 1 μg, meropenem (MEM) 10 μg, kanamycin (KAN) 30 μg, cefoxitin (FOX) 30 μg, sulfamethoxazole (SXM) 25 μg, and ceftazidime (CAZ) 30 μg [7]. *Klebsiella pneumoniae* ATCC13883 and *Staphylococcus aureus* ATCC25923 were used as the positive controls. The diameter of the zone of inhibition was measured, and the results were interpreted according to the breakpoint criteria established by the Clinical and Laboratory Standards Institute (CLSI).

Moreover, the pathogenic isolates exhibiting a detectable phenotypic profile for antibiotic resistance were subjected to resistance genotypic profiling. Specifically, 18 ARGs and two class 1 integrons (*intI1* and *intI1-V*) were examined in these isolates, following an experimental procedure previously described [7,13]. Briefly, purified genomic DNA of all isolates was used as the PCR template for amplification with oligonucleotide primers described in [7,28]. The PCR reaction mixture contained 0.4 µM of each primer, 1 ng/µL genomic DNA template, 0.06 U/µL of Grisp Taq DNA Polymerase, 1X Taq PCR Buffer, and 200 µM of dNTPs. The PCR reactions were carried out under conditions previously described [7,28]. The quality of extracted genomic DNA was assessed by the successful amplification of 16S rDNA fragments.

## 5. Conclusions

The environmental fate of plastic debris and their role in transmitting pathogenic bacteria and antibiotic resistance genes to humans and animals is still poorly understood, as there is only limited scientific evidence available. Our study investigates the colonization of multidrug-resistant (MDR) bacteria on four different plastic surfaces and evaluated the role of plastics in disseminating antibiotic resistance genes (ARGs) in an aquatic ecosystem. Furthermore, our study delves into the potential utilization of plastic materials as tools for monitoring the presence of pathogenic bacteria and associated antibiotic resistance genes (ARGs) in aquatic ecosystems. This approach offers a simple, non-invasive means of surveillance, bypassing the need for large-scale raw water sampling to detect the prevalence of these microorganisms in aquatic ecosystems.

The detection of complex resistance patterns and the identification of antibiotic resistance genes against tetracycline, chloramphenicol, gentamicin, meropenem, and kanamycin in *Klebsiella* and *Exiguobacterium* species underscores the pivotal role of plastic debris in facilitating the colonization and dissemination of antibiotic-resistant bacteria (ARB) in freshwater ecosystems. These findings call attention to the potential of different plastic substrates to act as underestimated trafficking agents for antibiotic-resistant bacteria, posing a significant threat to global health. The results of this work reveal critical insights into the prevalence of opportunistic human pathogens and antibiotic-resistant bacteria on plastic surfaces in freshwater bodies, emphasizing the need to take urgent actions to reduce the release of pollutants into aquatic environments and to control the spread of antibiotic resistance. Moreover, this study highlights the need to develop and implement new, efficient, and reliable strategies to mitigate the environmental burden of antibiotic resistance, and provides a foundation for future research, based on a rigorous combination of microbiological and genetic analysis. The present research contributes to identifying the drivers for the transmission of antibiotic resistance in aquatic environments. These are critical components of understanding and managing the resistance crisis and taking action to mitigate human exposure risks.

## Figures and Tables

**Table 1:** Summary of bacterial isolates recovered from four plastic polymers. Different gray shading highlights clusters of bacteria recognized by the API kit method. The mismatches occurring between the two identification methods are emphasized in bold.

Strain ID	Biochemical Identification	Molecular Identification	Plastic Polymer	Accession Number
BL70	*Staphylococcus captis*	***Lysinibacillus sphaericus***	Polypropylene	PP496519
BL71	*Staphylococcus haemolyticus*	***Lysinibacillus fusiformis***	Polystyrene	PP496520
BL72	*Staphylococcus xylosus*	***Exiguobacterium acetylicum***	Polystyrene	PP496532
BL73	*Staphylococcus xylosus*	***Exiguobacterium acetylicum***	Polystyrene	PP496533
BL74	*Staphylococcus lentus*	***Exiguobacterium acetylicum***	Polystyrene	PP496534
BL75	*Staphylococcus sciuri*	***Lysinibacillus boronitolerans***	Polystyrene	PP496524
BL76	*Staphylococcus sciuri*	***Lysinibacillus sphaericus***	Styrene acrylonitrile resin	PP496522
BL77	*Staphylococcus sciuri*	***Exiguobacterium acetylicum***	Styrene acrylonitrile resin	PP496535
BL78	*Staphylococcus sciuri*	***Exiguobacterium acetylicum***	Styrene acrylonitrile resin	PP496536
BL79	*Staphylococcus aureus*	***Lysinibacillus macroides***	Styrene acrylonitrile resin	PP496521
BL80	*Staphylococcus aureus*	***Lysinibacillus boronitolerans***	Styrene acrylonitrile resin	PP496525
BL81	*Staphylococcus aureus*	***Lysinibacillus sphaericus***	Styrene acrylonitrile resin	PP496523
BL82	*Staphylococcus aureus*	***Lysinibacillus fusiformis***	Styrene acrylonitrile resin	PP496526
BL83	*Klebsiella pneumoniae*	*Klebsiella pneumoniae*	Polystyrene	PP497002
BL84	*Klebsiella pneumoniae*	*Klebsiella pneumoniae*	Polystyrene	PP497003
BL85	*Klebsiella pneumoniae*	*Klebsiella pneumoniae*	Styrene acrylonitrile resin	PP497004
BL86	*Klebsiella oxytoca*	*Klebsiella oxytoca*	Polystyrene	PP497005
BL87	*Klebsiella oxytoca*	*Klebsiella oxytoca*	Styrene acrylonitrile resin	PP497006
BL88	*Klebsiella oxytoca*	***Klebsiella michiganensis***	Polystyrene	PP497007
BL89	*Klebsiella oxytoca*	***Klebsiella michiganensis***	Polystyrene	PP497001

**Table 2:** The identification of antibiotic resistance genes (ARGs) and genes that encode for class 1 integrons in plastic-associated bacteria was performed by targeted DNA amplification.

Bacterial Isolates	Antibiotic Resistance Genes																			
	*intI1-V*	*intI-1*	*blaCTX-M*	*blaSHV*	*blaTEM*	*mecA*	*cmlA1*	*cmx(A)*	*acrA*	*acrB*	*acrF*	*acrR*	*adeA*	*tetA*	*tetB*	*tetM*	*tetW*	*sul1*	*sul2*	*sul3*
*Lysinibacillus* spp. (*n* = 15)	8/15	3/15	4/15	7/15	8/15	12/15	0/15	0/15	0/15	5/15	7/15	0/15	5/15	3/15	0/15	0/15	9/15	10/15	0/15	0/15
*Exiguobacterium* spp. (*n* = 7)	5/7	0/7	0/7	3	4/7	5/7	0/7	3/7	0/7	4/7	2/7	1/7	3/7	2/7	0/7	0/7	2/7	0/7	0/7	0/7
*Klebsiella* spp. (*n* = 9)	5/9	6/9	6/9	8/9	7/9	0/9	0/9	4/9	0/9	7/9	5/9	4/9	7/9	6/9	0/9	0/9	5/9	5/9	5/9	0/9

## Data Availability

The data are contained within the article.
